# Children and young people’s body mass index measures derived from routine data sources: A national data linkage study in Wales

**DOI:** 10.1371/journal.pone.0300221

**Published:** 2024-05-10

**Authors:** Lucy J. Griffiths, James Rafferty, Richard Fry, Helen Daniels, Carol Dezateux, Nicola Firman, Theodora Pouliou, Gareth Stratton, Amy Mizen, Ronan A. Lyons, Alan Watkins, Jo Davies, Rowena Bailey

**Affiliations:** 1 Swansea University Medical School, Swansea, United Kingdom; 2 Wolfson Institute of Population Health, Queen Mary University of London, London, United Kingdom; 3 Research Centre in Applied Sports, Technology, Exercise and Medicine, Swansea University, Swansea, United Kingdom; University of Montenegro: Univerzitet Crne Gore, MONTENEGRO

## Abstract

**Background:**

Routine monitoring of Body Mass Index (BMI) in general practice, and via national surveillance programmes, is essential for the identification, prevention, and management of unhealthy childhood weight. We examined and compared the presence and representativeness of children and young people’s (CYPs) BMI recorded in two routinely collected administrative datasets: general practice electronic health records (GP-BMI) and the Child Measurement Programme for Wales (CMP-BMI), which measures height and weight in 4-5-year-old school children. We also assessed the feasibility of combining GP-BMI and CMP-BMI data for longitudinal analyses.

**Methods:**

We accessed de-identified population-level GP-BMI data for calendar years 2011 to 2019 for 246,817 CYP, and CMP-BMI measures for 222,772 CYP, held within the Secure Anonymised Information Linkage Databank. We examined the proportion of CYP in Wales with at least one GP-BMI record, its distribution by child socio-demographic characteristics, and trends over time. We compared GP-BMI and CMP-BMI distributions. We quantified the proportion of children with a CMP-BMI measure and a follow-up GP-BMI recorded at an older age and explored the representativeness of these measures.

**Results:**

We identified a GP-BMI record in 246,817 (41%) CYP, present in a higher proportion of females (54.2%), infants (20.7%) and adolescents. There was no difference in the deprivation profile of those with a GP-BMI measurement. 31,521 CYP with a CMP-BMI had at least one follow-up GP-BMI; those with a CMP-BMI considered underweight or very overweight were 87% and 70% more likely to have at least one follow-up GP-BMI record respectively compared to those with a healthy weight, as were males and CYP living in the most deprived areas of Wales.

**Conclusions:**

Records of childhood weight status extracted from general practice are not representative of the population and are biased with respect to weight status. Linkage of information from the national programme to GP records has the potential to enhance discussions around healthy weight at the point of care but does not provide a representative estimate of population level weight trajectories, essential to provide insights into factors determining a healthy weight gain across the early life course. A second CMP measurement is required in Wales.

## Introduction

The global rise in childhood obesity is a serious, long-term public health challenge and one that has been exacerbated by the COVID-19 pandemic [1]. Widening inequalities exacerbate this trend with the most socioeconomic deprived populations disproportionately affected with higher prevalence of obesity [2]. Population-based overweight and obesity monitoring/surveillance systems are critical for monitoring trends in childhood obesity and related behaviours to inform obesity planning strategies and to determine the overall effect of specific government or other public health initiatives [3].

The Child Measurement Programme (CMP) for Wales, and similarly the National Child Measurement Programme (NCMP) in England, provide robust public health surveillance data on the weight status of school-aged children, gathered to assess levels of childhood obesity at local and national levels. All four UK nations collect systematic and standardised height and weight measures upon entry to primary school at age 4–5 years, and, in England, upon leaving primary school at age 10–11 years [4–7].

Anthropometric measurements of height and weight are also available within National Health Service (NHS) general practice electronic health records (GP-EHRs); however, they are not taken routinely and–in adults–it has been shown that they are most commonly measured in individuals with a BMI considered under- or overweight, or in relation to a clinical diagnosis [8,9]. As such, missing BMI records are unlikely to be missing at random. Although analysis of general practice (GP) records has been carried-out to monitor obesity trends in CYP [10], concern has been raised about the representativeness and accuracy of measurements taken in primary care when compared with NCMP measures [11]. Firman et al [11] recommend linkage of information from both sources to better identify unhealthy weights and their relation to health outcomes.

There is a need to understand the representativeness of CYP in Wales with Body Mass Index (BMI) records in linked administrative data sources. Further evidence on the completeness of CYP’s BMI recorded in general practice EHRs (GP-BMI records), and their potential to be used in combination with information from the CMP (CMP-BMI), could enable BMI trajectories to be explored and the impact of childhood exposures (such as environment determinants) on growth patterns to be examined.

We therefore aimed to a) evaluate the proportion of CYP in Wales with a GP-BMI record and the distribution of GP-BMI records by child demographic and socio-economic characteristics, as well the trends of these proportions over time; b) evaluate the representativeness of primary care records of childhood weight by comparing the GP-BMI distributions with those derived from the CMP; and c) quantify the proportion of children with a CMP-BMI measure and a follow-up GP-BMI record from a later date (to assess the feasibility of using the combined data sources for longitudinal analysis), and the representativeness of these measures.

## Methods

### Study design & population

This is a population level, retrospective, observational study which utilised routinely collected data, combining cross-sectional data from the CMP and longitudinal EHRs relating to childhood BMI measures. We worked with two separate cohorts. For our first objective, we included all CYP under the age of 18 years resident at a Welsh address between 1^st^ January 2011 and 31^st^ July 2019, registered to a Secure Anonymised Information Linkage (SAIL) providing GP practice (see below). For our second objective, we derived the cohort as above with the additional constraint that CYP also had a CMP-BMI measure.

### Data sources and access

All data were analysed and accessed via the SAIL Databank, hosted at Swansea University [12,13]. The SAIL Databank is a privacy-protecting Trusted Research Environment which houses routinely collected EHRs and administrative data sources relating to the population of Wales, United Kingdom. For each data source within the SAIL Databank, personal identifiable data has been removed and replaced with an anonymised linkage field (ALF) for each person to enable linkage of records from various sources (the anonymisation and linkage methodology is described elsewhere) [12–14]. All data within the SAIL Databank are treated in accordance with the Data Protection Act 2018 and comply with the General Data Protection Regulation, 2016.

The following data sources were used in this study:

The Welsh Longitudinal General Practice dataset is derived from records of patient interactions with NHS primary care services in Wales. It includes records of events such as consultations with GPs or nurses, medication and treatment prescriptions, clinical tests and physiological measurements. The GP data in SAIL covers approximately 83% of the population of Wales [15].

The National Community Child Health Database (NCCHD) brings together data from local child health system databases held by NHS organisations and includes information from birth registrations, child health examinations and vaccinations. Since 2011, heights and weights of children in reception class of state-maintained schools, collected via the CMP, have also been entered on the CMP module within NCCHD. Parents/carers of the children are all given the opportunity to opt their children out of the measurement programme, although participation remains high; for example, participation in the programme in 2018/19 was 93.4% [16]. These CMP records (from school years 2011/12-2018/19) are available within SAIL and were used for this study.

The Welsh Demographic Service Dataset (WDSD) contains address records and demographic information for people accessing NHS services in Wales and registered to a Welsh address. WDSD was used to derive age, sex, and socio-economic deprivation quintile for the cohort. WDSD was also used to calculate population denominators.

### Measures

#### BMI status

We obtained anthropometric data, including actual dates of measurement, from the GP data. Recorded BMI measurements were obtained for all study CYP. In addition, measured height and weight values were also extracted to calculate BMI directly. We required that height and weight be measured on the same day to calculate BMI. Heights (h) and weights (w) were converted to m and kg respectively and BMI was calculated using the formula $BMI=\frac{w}{h^{2}}$. The Read codes (a coded thesaurus of clinical terms in the NHS) used to extract these height, weight and BMI values in the GP data were compiled from academic articles on similar research [9–11] and from reference tables held within the SAIL Databank [12].

BMI measurements recorded in the CMP are carried out in a standardised way by trained school health team members across Wales and were used directly.

We calculated Z-scores for each BMI measurement; those more than five standard deviations away from the mean were discarded as outliers. We applied this conservative cut-off to remove only the most extremely implausible records since the purpose of this study was to compare quality of GP data. Z-scores were converted to sex- and age-specific BMI centiles using the LMS method (which produces normalised growth centile standards) [17,18].

BMI status was derived from centile thresholds using UK1990 clinical reference standards [19], resulting in four mutually exclusive groups: “underweight” (BMI <second centile), “healthy weight” (≥second to <91st centile), “overweight” (≥91st to <98th centile), or “very overweight” (≥98th centile) (sometimes referred to as obese).

#### Covariates

Age (in years) was calculated at the date of the GP-BMI measure using week of birth from the WDSD. Where individuals had more than one GP-BMI measure, the first record was used for reporting descriptive statistics. Age groups of three-year bands starting at age 4 were used, since the band 4–6 included most children measured as part of the CMP. Sex was also obtained from the WDSD and is a dichotomous variable representing males and females. The WDSD also contains the Lower layer Super Output Area (LSOA) code of the address registered as the child’s residence at the date of the recorded BMI measure. We used the LSOA codes derived from the 2011 Census.

Welsh Index of Multiple Deprivation (WIMD) is the Welsh Government’s official socio-economic deprivation measure for small areas in Wales. This study used WIMD 2019 [20]. Since our study is related to health outcomes, the health domain was removed from the WIMD score as advised in the guidance [20]. At the domain and overall level, the 1909 LSOAs are ranked in order of deprivation, with 1 being the most deprived and 1909 being the least deprived. Quintiles were ranked 1 to 5, with 1 being the most deprived group and 5 the least deprived.

### Statistical analysis

The overall proportion of CYP with BMI measures in GP data was derived as a percentage of the total number of CYP registered to a SAIL providing GP practice on the 1^st^ of July for each study year. This date was chosen to align with the school census date used by the CMP. Descriptive statistics of children’s characteristics are presented as distributions of measures by age, gender and deprivation across study years.

The representativeness of BMI measures in GP data was evaluated from analysis of derived Z-scores and comparing the distribution of Z-scores of 4–5-year-olds with the distributions derived from CMP data using a quantile-quantile (QQ) plot and a two sample Kolmogrov-Smirnov test. We interpreted an association as statistically significant where the p-value was less than 0.05.

We used logistic regression to evaluate if the likelihood of a follow-up measure recorded in GP data was associated with the child’s characteristics measured in the CMP. We exponentiated the model coefficients and report the adjusted odds ratio (OR) along with 95% confidence intervals (CI). We interpreted statistically significant associated where the CI did not include one.

### Ethics

This study was approved by SAIL Information Governance Review Panel (project 1001) in Wales. All data were anonymised (de-identified) prior to access and analysis. In accordance with Health Research Authority guidance [21], ethical approval is not mandatory for studies using only anonymised data and so was not obtained for this study.

## Results

### GP-BMI records

**Fig 1:**
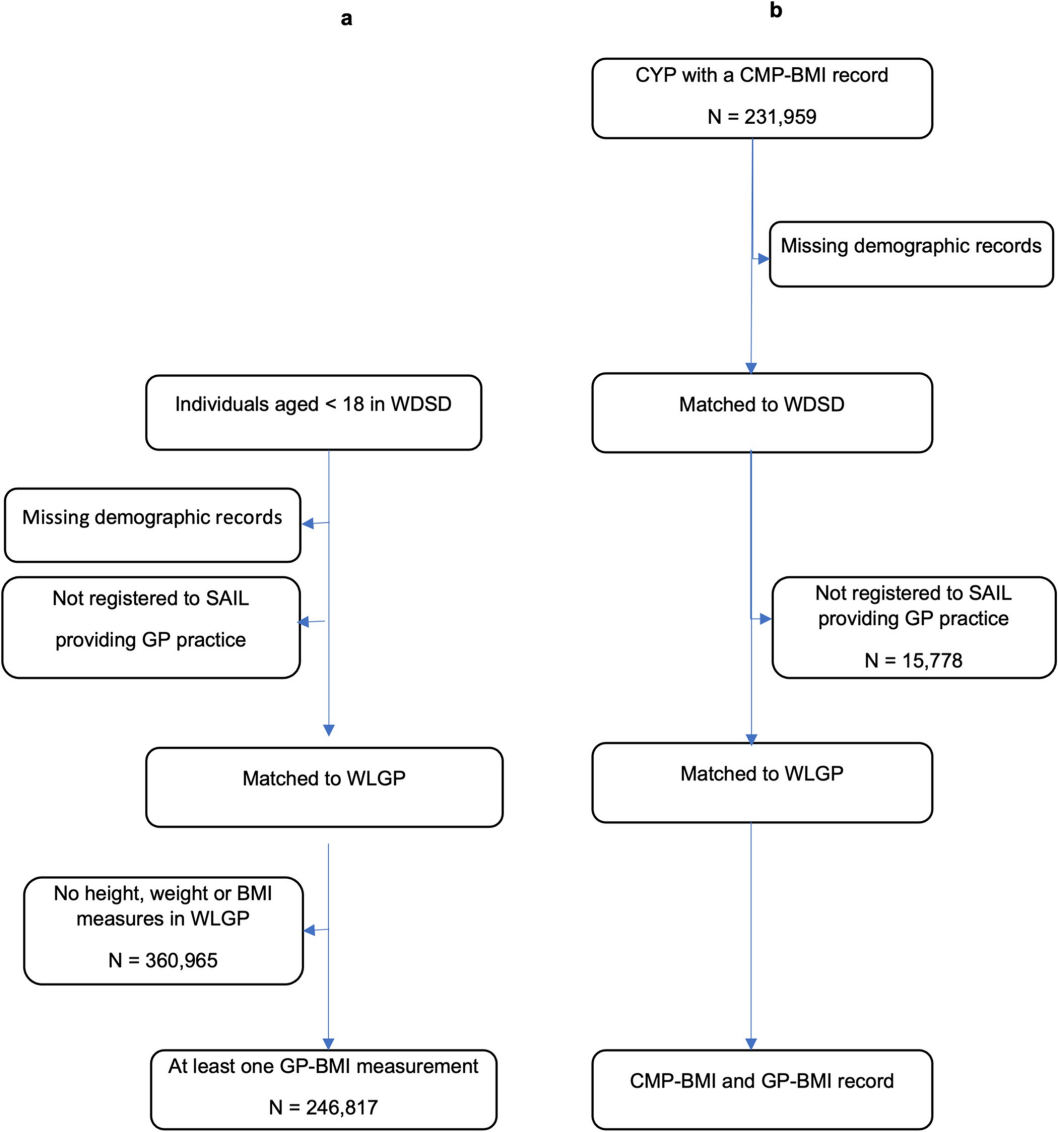
GP-BMI data cohort (a) and CMP and GP-BMI data cohort (b).

Between 2011 and 2019, there were a total of 776,491 unique individuals aged under 18 years and resident in Wales. We excluded 73,480 records with missing demographic data and, of those remaining, 607,782 (87%) were registered to a SAIL-GP during that period. Of those, 246,817 (41%) had height and weight data recorded on the same day or a recorded BMI–thus at least one GP-BMI record (Fig 1A).

People under 18 years of age with GP data coverage in SAIL were representative of the general population, with comparable distributions by age, sex and deprivation to those published by ONS (Tables 1 and S1). These data show slighter more males than females, an even spread of CYP by single year of age, and a high proportion living in the most deprived quintiles (more than 46% of all CYP in Wales living in the two most deprived quintiles).

**Table 1:** Demographic distributions (number and percentage) for: i) persons with any GP data, defined as any interaction with a SAIL contributing primary care service over the study period, ii) of those, persons with GP data AND a measure of BMI, where BMI is either a direct record of BMI or a measurement of weight and height on the same day over the study period and iii) the total number of recorded measurements of BMI from all persons in primary care over the study period.

	Persons with GP record (with or without any BMI measure)	Persons with GP record AND at least one GP-BMI measurement	Total number of GP-BMI measurements
Total persons < 18 years	486,660 (100%)	246,817 (100%)	623,051 (100%)
**Sex**			
Males	249,489 (51.3%)	113,090 (45.8%)	284938 (45.7%)
Females	237,171 (48.7%)	133,727 (54.2%)	338113 (54.3%)
**Age (in years at date of measure)**			
<1	21,755 (4.5%)	70,859 (20.7%)	98,123 (15.7%)
1	24,685 (5.1%)	3,716 (1.5%)	11,560 (1.9%)
2	24,922 (5.1%)	4,272 (1.7%)	12,020 (1.9%)
3	26,065 (5.4%)	8,834 (3.6%)	20,335 (3.3%)
4	26,831 (5.5%)	6,605 (2.7%)	20,335 (3.3%)
5	27,069 (5.6%)	7,633 (3.1%)	20,141 (3.4%)
6	27,647 (5.7%)	7,655 (3.1%)	21,171 (3.4%)
7	29,332 (6.0%)	7,931 (3.2%)	22,953 (3.7%)
8	29,347 (6.0%)	8,116 (3.3%)	24,509 (3.9%)
9	28,843 (5.9%)	8,359 (3.4%)	25,525 (4.1%)
10	28,779 (5.9%)	8,407 (3.4%)	25,671 (4.1%)
11	29,428 (6.1%)	8,686 (3.5%)	27,142 (4.4%)
12	28,607 (5.9%)	9,275 (3.8%)	28,623 (4.6%)
13	27,582 (5.7%)	10,645 (4.3%)	31,736 (5.1%)
14	27,148 (5.6%)	13,140 (5.4%)	37,132 (6.0%)
15	26,647 (5.5%)	17,774 (7.2%)	51,316 (8.2%)
16	26,383 (5.4%)	22,147 (9.0%)	68,442 (11.0%)
17	25,590 (5.3%)	22,763 (9.2%)	79,687 (12.8%)
**Deprivation (WIMD quintile)**			
1 Most Deprived	124,919 (25.7%)	60,038 (24.3%)	157,778 (25.3%)
2	100,574 (20.7%)	51,993 (21.1%)	136,748 (21.9%)
3	85,237 (17.5%)	45,028 (18.2%)	113,108 (18.1%)
4	83846 (17.23%)	44455 (18.0%)	110,632 (17.8%)
5 Least Deprived	92084 (18.92%)	45303 (18.4%)	104,785 (16.8%)

CYP with a GP-BMI record were more likely to be female and infants or older adolescents compared to all those with GP data (Table 1). The distribution of CYP with GP-BMI measures by deprivation quintile was representative of all those registered to a SAIL-GP.

Overall, the proportion of CYP with a GP-BMI record did not change over the study period (2011–2019), although the proportion with a GP-BMI at age 4 or 5 increased, with 4.2% having a GP-BMI measurement in 2011 and 5.9% having one in 2019 (S1 Fig).

### Representativeness of GP-BMI records

For the CMP cohort, there were 222,772 CYP (109,042, 49.0% female) measured in total from 2011–2019 with complete demographic information available. Of these, 206,994 CYP were registered to a SAIL providing GP during the same period: 31,521 (15.2%) had at least one (follow-up) GP-BMI record before the age of 18 years (Fig 1B).

Over the study period, 81.2% of children measured in the CMP had healthy weight, 11.1% with a BMI considered overweight and 6.9% of children very overweight. Less than 1% of children (0.8%) had a BMI classified as underweight.

**Fig 2:**
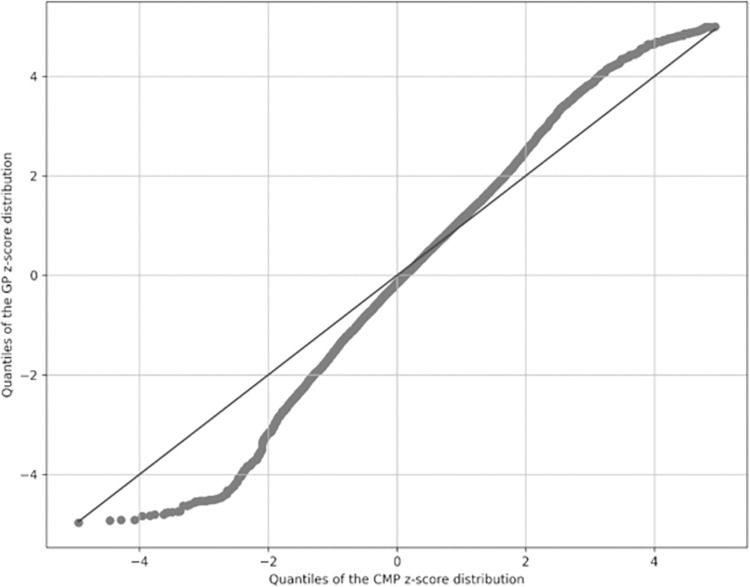
QQ plot comparing distribution of z-scores in the CMP (x-axis) and in primary care (y-axis).

When comparing CMP-BMI measures with GP-BMI measures (for 4- and 5-year-olds only) (n = 20,440), we found higher proportions of children categorised as underweight and very overweight in GP-BMI measurements. Only 72.8% of children’s GP-BMI measurements were considered a healthy weight, while 11.9% overweight, 11.7% very overweight and 3.6% underweight. The distribution of z-scores from both data sources represented as a QQ plot (Fig 2) shows deviations at both tails of the distributions, further indicating that CYP with underweight or overweight/very overweight BMI measures were more represented in GP-BMI measures compared to the CMP data.

The two sample Kolmogrov-Smirnov test statistic comparing these distributions was 0.07 (p<0.001) indicating that the sample of GP-BMI measures and the CMP BMI measures were not drawn from the same underlying distribution and as such the GP-BMI data were not representative of the population.

### Follow-up GP-BMI records for children in the CMP

**Fig 3:**
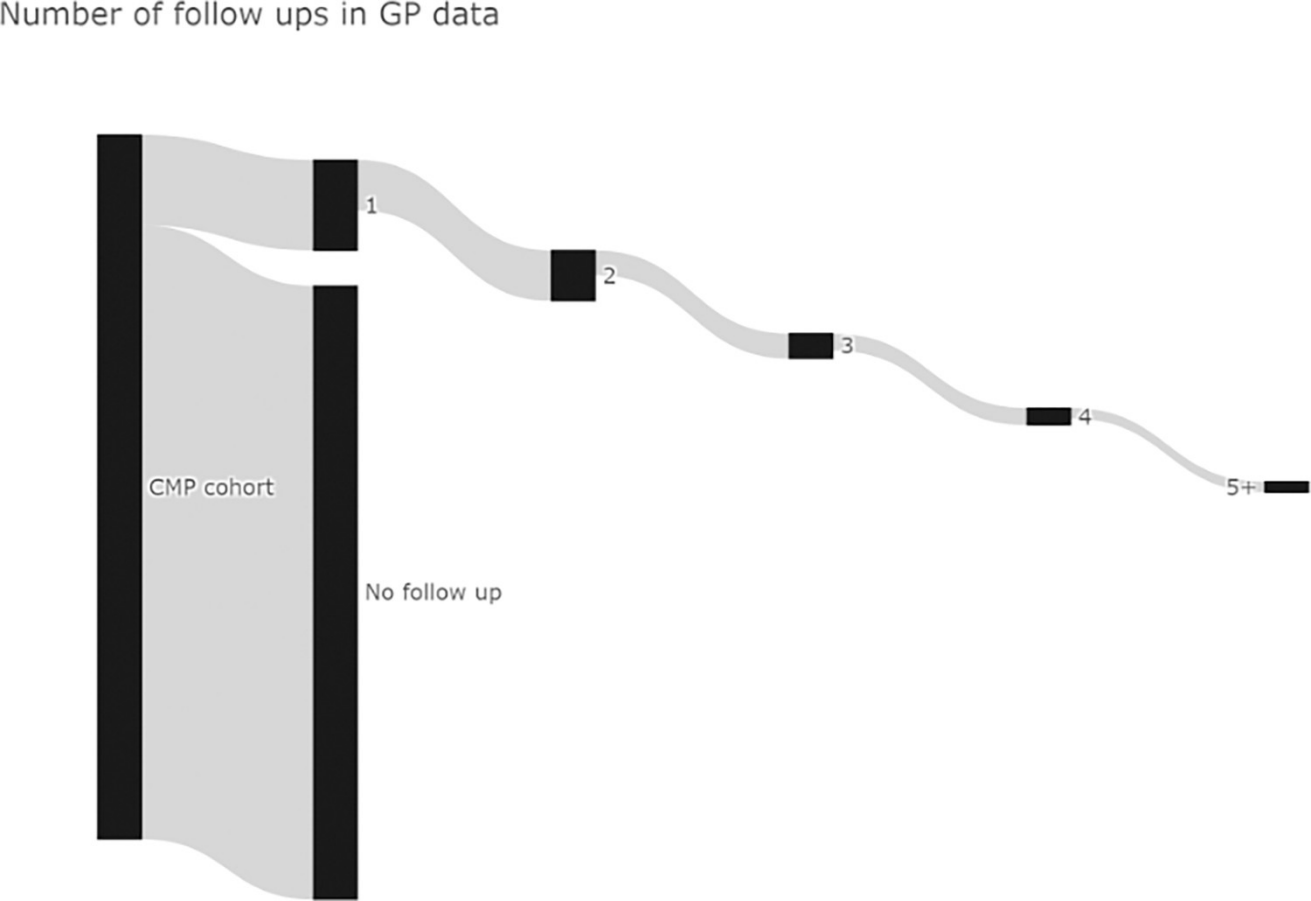
BMI measurements and primary care follow-up.

Fig 3 illustrates children with a BMI measurement from the CMP cohort (left most bar) and the number of follow-up measurements of their BMI they had in primary care before the age of 18. 13,931 (6.7%) CYP had only one follow-up measurement and 3,694 (1.8%) had five or more follow-up GP-BMI measurements.

**Fig 4:**
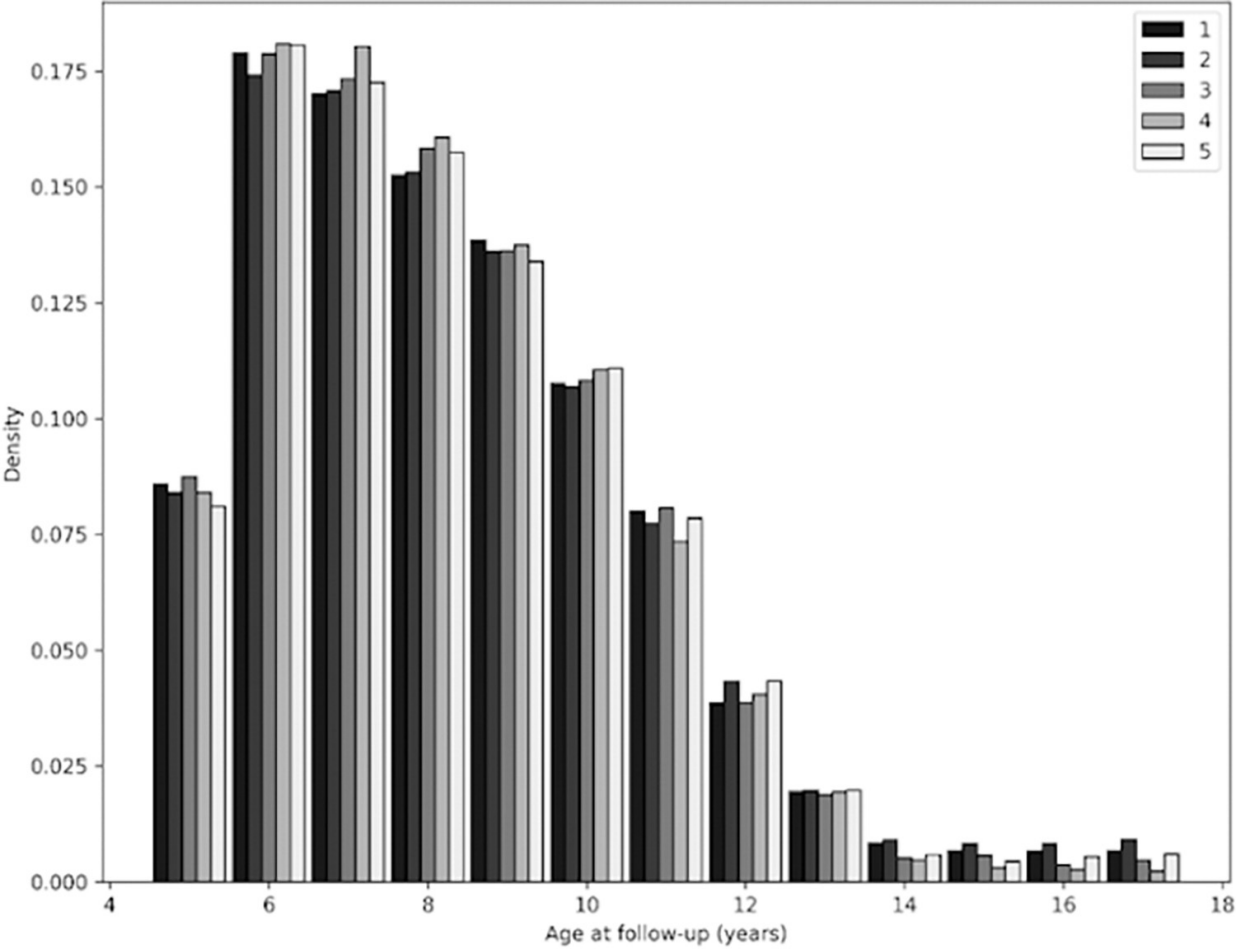
The number of follow-ups in primary care by deprivation and age. Each colour represents a different deprivation quintile, with quintile 1 being the most deprived and quintile 5 being the least deprived.

The mean age of a child’s initial BMI measure in the GP data was 8.3 years, while the mean age of all measurements performed in primary care was 10.1 years. The number of follow ups falls rapidly around the time children start attending secondary school at age 11 years, and there was little variation in number of follow ups by deprivation quintile (Fig 4).

Results from the logistic regression show that the factor most strongly associated with the presence of a follow up BMI measure in GP data was the BMI category measured in the CMP (Table 2). Children considered underweight were 87% more likely to have a follow-up GP measure (OR = 1.87, 95% Confidence Interval (CI) = 1.66, 2.10) and children considered very overweight, or overweight were 70% (OR = 1.70, 95% CI = 1.63, 1.77) and 17% (OR = 1.17, 95% CI = 1.13, 1.22) more likely respectively, compared to those with a healthy weight measured in the CMP. Follow-up measures were also increasingly more likely with higher levels of deprivation, with children from the two most deprived quintiles being 25% (OR = 1.25, 95% CI = 1.20, 1.30) and 26% (OR = 1.26, 95% CI = 1.21, 1.26) more likely to have a GP-BMI measure than those in the least deprived quintile. GP-BMI measures were 19% less likely to be recorded for females (OR = 0.81, 95% CI = 0.79, 0.83) compared to males.

**Table 2:** Factors associated with GP follow-up measures.

Predictor	Odds Ratios (95% CI)	p-value
**Deprivation (WIMD quintile)**		
1 Most Deprived	1.25 (1.20, 1.30)	<0.001
2	1.26 (1.21, 1.31)	<0.001
3	1.09 (1.04, 1.13)	<0.001
4	1.04 (1.00, 1.09)	0.06
5 Least Deprived (baseline)	1	
**CMP-BMI**		
Underweight	1.87 (1.66, 2.10)	<0.001
Healthy Weight (baseline)	1	
Overweight	1.17 (1.13, 1.22)	<0.001
Very overweight	1.70 (1.63, 1.77)	<0.001
**Sex**		
Male (baseline)	1	
Female	0.81(0.79, 0.83)	<0.001

## Discussion

Less than half (41%) of the CYP with GP data had a GP-BMI record. Those who were female, infants or adolescent were more likely to have a GP-BMI record, compared to all those with GP data. Deprivation quintiles were similarly distributed between those with and without GP-BMI records. Eighteen percent of children measured in the CMP had a BMI categorised as overweight or very overweight and 0.8% as underweight, compared to 23.6% and 3.6%, respectively, of children with a GP-BMI measurement (4–5-year-olds only). GP-BMI measures were therefore not representative of the population of children within this age group when compared to the underlying distribution of those measures from the CMP: those with below or above healthy BMIs are more likely to be measured by their general practitioner and hence had a BMI record.

For our purpose of assessing the feasibility of using administrative data to supplement the CMP for longitudinal analysis of child growth trajectories, we quantified the proportion of children with both a CMP measure of BMI and a follow-up GP-BMI record during later childhood, and report that only 15.2% of the CMP cohort had at least one follow-up measure recorded in their EHRs. Children from the most deprived areas, males, and those who had a BMI categorised as underweight, overweight, or very overweight were more likely to have a follow-up measure.

This is the first study to report on the completeness and representativeness of children’s BMI measurements based on GP-EHRs in Wales. To our knowledge, it is also the first to link the GP-EHRs to CMP data in Wales. Similar work has been carried out in England. Firman et al. [11] studied the completeness and accuracy of children’s BMI in primary care records. They linked NCMP records from 5-year-olds and 11-year-olds attending state schools in inner London to GP-EHRs and estimated adjusted odds (aOR) of at least one GP-BMI record by sex, ethnic background, area-level deprivation, weight-status and long-term conditions. This study reported that 10.5% of 5-year-olds in east London had at least one GP-BMI record which was lower than the 15.2% that we found in this study for 5-year-olds in Wales.

Regarding the follow-up measures of the CMP in the GP-BMI records, we reported that children from the most deprived areas, males, and those with a BMI categorised as underweight, overweight or very overweight were more likely to have a follow-up measure. This finding is similar to that of Firman et al [11] where children were more likely to have at least one GP-BMI recorded if they were: male; from South Asian ethnic backgrounds; living in the most deprived areas; recorded as having a long-term condition and categorised as underweight or very overweight in the NCMP. The latter study additionally explored the quality of GP-BMI records, and whilst they found these to be good, they also identified wide within-child variation between BMI values recorded by the GP and those recorded in the NCMP.

Using the UK1990 clinical reference standards, we report that 11.1% and 6.9% of 5-year-olds were classified as overweight or very overweight compared to 11.9% and 11.7%, respectively, of children with a GP-BMI measurement. Firman et al found that 9.6% and 8.2% of 5 years old were overweight or very overweight compared to 7.9% and 18.2%, respectively, of children with a GP-BMI measurement. Both studies indicate that GP-BMI measures were not representative of the underlying distribution of those obtained from the CMP: those with below or above healthy BMIs are more likely to be measured by their general practitioner and hence had a BMI record. Kelly et al. [22] found that children who were underweight or obese at the age of 5 years had significantly higher rates of GP appointments compared to the healthy weight children. This finding was also supported by Dezateux et al. [23] who reported that consultation rates were higher following NCMP measurements for children who were classified underweight or obese.

Internationally, regular screening for unhealthy weight status in children is recommended, although there is little evidence regarding appropriate screening intervals [24]. Within the UK, the National Institute for Health and Care Excellence recommends that GPs refer adults with excess weight to weight management services; however, it is not clear whether similar frameworks for children exist. Recording of children’s heights, weights and BMI is not routine in general practice: GPs are advised to use clinical judgement to decide when to measure a child or young person’s height and weight.

Our novel linkage of CMP data to EHRs in Wales was enabled through the SAIL Databank. In doing so, we have assessed the feasibility and value of using routine collected national primary care data to augment cross-sectional annual data collections of childhood BMI measures in the CMP to facilitate longitudinal analysis of BMI trajectories. We have found non-representativeness of GP derived BMI measures for children aged between 4–5 years. As there is no nationally representative collection of BMI for older children in the CMP, we assume the non-representativeness identified in this age group is applicable in older age groups. Furthermore, only a small proportion of the CMP cohort had a follow-up measure in GP data.

However, we acknowledge the following limitations of our work to date. Firstly, not all GP practices in Wales had agreed to share data with the SAIL Databank for research at time of study, with the coverage currently being 83% of the population of Wales and 80% of GP practices in Wales; nevertheless, the GP practices currently signed up to SAIL are considered to be representative of the population in terms of patient demographic and socio-economic characteristics [15]. Further, by virtue of the design of the CMP, we have only captured children in state-maintained primary schools, and those attending schools in Wales. Finally, we have only explored several socio-demographic factors, further work could explore representation of GP-BMI by health characteristics of the child and/or their family.

The latest all-Wales data published by the CMP in Wales was from 2018/19, which showed a rise in childhood obesity over the previous six years, with Welsh children more likely to be living with overweight/very overweight weight status at reception age than children in Scotland or England. More recent statistics from 2019/2020 are limited, as data collection was interrupted due to school closures during the pandemic. Measures obtained in two Health boards across Wales represent a significant rise in excess weight [6].

The Healthy Weight Healthy Wales delivery plan for 2022–2024 [25] highlights a commitment to expand the CMP. This is currently the only reliable population-level coverage of BMI measures among children across Wales; measurements are taken by staff trained in a standardised way, using equipment that is calibrated annually [16]. However, as it is currently limited to 4/5-year-olds (in state-maintained schools), extending it to include a follow-up measure at the end of primary school and/or within secondary school would enable children’s BMI trajectories to be tracked to monitor change. National analysis of weight status change between the first and final years of primary school using NCMP data [26], provides evidence into how it can be tracked, as well as the flow of children from a healthy to unhealthy weight status, contributing to the higher prevalence of obesity at the end of primary school. A pilot surveillance project examining heights and weights of children attending year 4 (children aged 8 to 9) in two local authorities in Wales supported extension of the CMP [27]. Further, Jarvis et al [28]. call for an additional follow-up measure based on their work examining adiposity levels at ~10 years and predicting future risk upon entering young adulthood. However, it is important to note that the CMP is not considered a screening programme, and recently published work on household member’s experiences of the programme in England suggests the need for it to consider potential social, emotional and moral harms for participating children and their parents [29]. More consideration needs to be given to when and how to intervene with additional support to promote healthy weight for those categorised as overweight either at baseline and/or follow-up.

National surveillance data in primary care can be used to identify and recruit children with excess weight and their parents to participate in school and general practice-based research and/or interventions, and to inform families of children’s measurements [30]. Our data thus suggests the utility of available measurements for such a purpose, but not for population level health research given the lack of representativeness of measures.

This study focused on the availability and representativeness of BMI measures; for those interested in secular trends in BMI and inequalities, weight gain and height growth should be explored separately using available measures within GP EHRs [31]. Further work is also needed to examine the variation in availability of measurements by clinical diagnoses.

In conclusion, childhood weight and factors that affect healthy weight gain in the early years are a major public health concern given the rising prevalence of excess weight. Research which provides a better understanding of the preventable wider determinants which lead to childhood health inequalities are essential to inform policy aimed at tackling this issue. To do so, population-based monitoring of weight status throughout childhood is required. Records of childhood weight status extracted from general practice are not representative of the population and are biased with respect to weight status. BMI information from the CMP, from EHRs and linkage of information from both data sources may provide a more comprehensive set of measures but will not provide a representative estimate of population level BMI trajectories, essential to provide insights into factors determining a healthy weight gain across the early life course. Repeat weight and height measurement as part of the national programme is therefore important and, as such, the CMP in Wales needs to be expanded to include a follow-up measure at the end of primary school and/or within secondary school [32].

## Supporting information

S1 Fig The proportion of 4- or 5-year-olds with a GP-BMI measurement by year. (TIF)

S1 Table Office for national statistics population profile. (DOCX)

## Abbreviations

ALF: anonymised linkage field
BMI: Body Mass Index
CMP: Child Measurement Programme
CI: Confidence Interval
CYP: children and young people
EHRs: electronic health records
GP: general practice
NHS: National Health Service
LSOA: Lower layer Super Output Area (LSOA)
NCCHD: National Community Child Health Database
NCMP: National Child Measurement Programme
OR: odds ratio
ONS: Office for National Statistics
SAIL: Secure Anonymised Information Linkage
WDSD: Welsh Demographic Service Dataset
WIMD: Welsh Index of Multiple Deprivation
