# Supplementary figures and images for: Children and young people’s body mass index measures derived from routine data sources: A national data linkage study in Wales
Source: PLoS One. 2024 May 10;19(5):e0300221. doi: 10.1371/journal.pone.0300221 (PMC11086882; doi:10.1371/journal.pone.0300221)

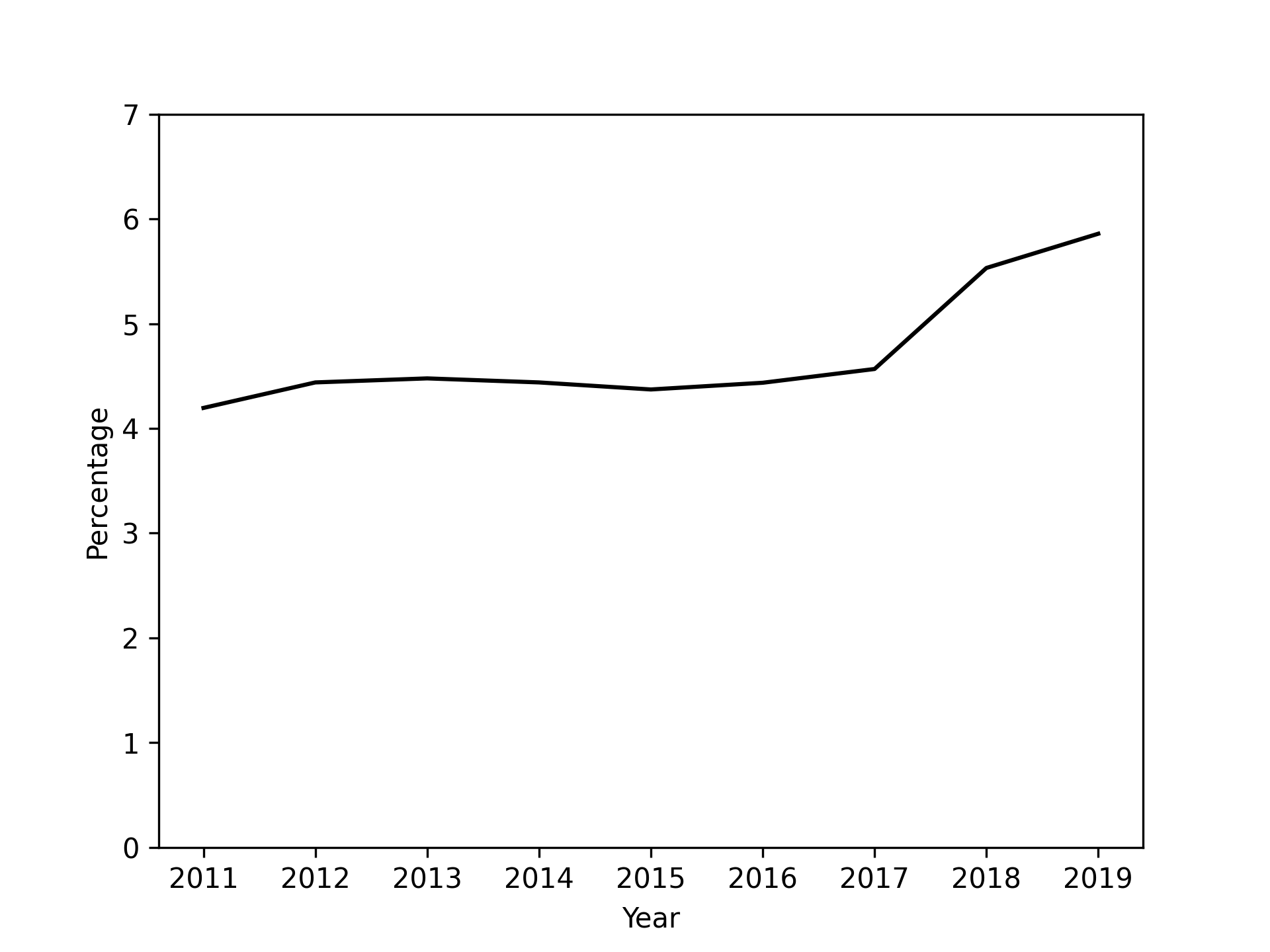

Supplement: S1 Fig — (TIF) [file pone.0300221.s001.tif]
