# Supplementary material for: Children and young people’s body mass index measures derived from routine data sources: A national data linkage study in Wales
Source: PLoS One. 2024 May 10;19(5):e0300221. doi: 10.1371/journal.pone.0300221 (PMC11086882; doi:10.1371/journal.pone.0300221)
Supplement: S1 Table — (DOCX) [file pone.0300221.s002.docx]

|  | **Number of persons in the ONS population** | **Proportion** |
| --- | --- | --- |
| **Total persons < 18 years** | 629,939 | 100.0% |
| **Sex** |  |  |
| Males | 323,069 | 51.3% |
| Females | 306,870 | 48.7% |
| **Age (years)** |  |  |
| 0 | 30,530 | 4.9% |
| 1 | 32,356 | 5.1% |
| 2 | 33,241 | 5.3% |
| 3 | 34,586 | 5.5% |
| 4 | 34,829 | 5.5% |
| 5 | 35,195 | 5.6% |
| 6 | 36,002 | 5.7% |
| 7 | 37,287 | 5.9% |
| 8 | 37,969 | 6.0% |
| 9 | 37,331 | 5.9% |
| 10 | 36,795 | 5.8% |
| 11 | 37,514 | 6.0% |
| 12 | 36,169 | 5.7% |
| 13 | 35,794 | 5.7% |
| 14 | 34,547 | 5.5% |
| 15 | 33,690 | 5.4% |
| 16 | 33,112 | 5.3% |
| 17 | 32,992 | 5.2% |
| **Deprivation (WIMD quintile)** | |  |
| 1 Most Deprived | 148,680 | 23.6% |
| 2 | 126,270 | 20.0% |
| 3 | 119,382 | 19.0% |
| 4 | 119,383 | 19.0% |
| 5 Least Deprived | 116,224 | 18.5% |
